# Zinc Ion Removal on Hybrid Pectin-Based Beads Containing Modified Poly(Methyl Methacrylate) Waste

**DOI:** 10.3390/molecules22122274

**Published:** 2017-12-20

**Authors:** Agata Jakóbik-Kolon, Adrianna Szybaj, Krzysztof Mitko, Joanna Bok-Badura

**Affiliations:** Faculty of Chemistry, Silesian University of Technology, Krzywoustego 6, 44-100 Gliwice, Poland; adusia.kat@wp.pl (A.S.); krzysztof.mitko@polsl.pl (K.M.); joanna.bok-badura@polsl.pl (J.B.-B.)

**Keywords:** zinc, sorption, hybrid sorbent, waste PMMA, pectin

## Abstract

A new hybrid sorbent in the form of round beads containing modified poly(methyl methacrylate) (PMMA) waste immobilized in pectin and crosslinked with calcium ions was prepared. A previously obtained and characterized powdered poly(methyl methacrylate)–based sorbent was used. Batch and column studies on the new material’s sorption-desorption properties were performed. Two kinetic models (pseudo-first- and pseudo-second-order) and three isotherms (Langmuir, Langmuir bisite and Freundlich) were used to describe the results. Breakthrough and elution curves were also obtained. Nitric, hydrochloric, and sulfuric acid of various concentrations were used in the desorption studies. Higher sorption affinity of zinc(II) ions to hybrid sorbent than to pectin alone, reflected by higher values of the Langmuir and Freundlich model parameters, was observed. The maximum sorption capacities, calculated based on the best-fitted models, were 50.2 mg/g (Langmuir bisite) and 42.2 mg/g (Langmuir) for hybrid and only pectin beads, respectively. The stripping of Zn ions using 0.1 M solutions of mineral acids was similarly effective in the case of both sorbents. The mass balance calculated for the column studies showed about 100% recovery of zinc in a sorption-desorption cycle. By applying the hybrid sorbent under the studied conditions it is possible to purify Zn in water to the level permitted by law and concentrate Zn(II) ions by about 60 times.

## 1. Introduction

Zinc and its compounds are present in the waste waters originating from various branches of industry and agriculture. Zinc usage is enormous—over 13 million tons are produced annually worldwide and consumed mainly as galvanizing products (e.g., strip mill carbon steel products for the construction and automotive industry), die-casting alloys, brass and castings (automotive products, electrical and hardware industries), products for roofing, oxides and other compounds (medical products, cosmetics, paints, batteries). Although Zn is an important element for living organisms in small amounts, especially for humans, its excess may cause serious problems in the brain, respiratory tract, gastrointestinal tract, and prostate [1,2] and is a serious environmental hazard (e.g., changes in the microbiological and biochemical properties of soil [3]). Therefore, depending on the country or waste origin, limits for Zn in wastewater dumped into the environment are prescribed by law, ranging from 1.5 to 2.61 mg/L [4,5,6]. Depending on the origin, the zinc concentration in wastewaters varies [7,8,9,10,11,12] from about 6 mg/L (rinsing water of degreasing and metal plating) [12] to 500 mg/L (from the electroplating industry) [7], but generally is below 100 mg/L [8,9,10,11,12]. Taking into account the limits, all of the wastewaters have to be purified. The most effective methods for these rather low concentrations of pollutants are membrane processes, adsorption, ion exchange or sorption, which blends adsorption processes with ion exchange. The proecological and economical approach is to use, as sorbents, waste materials, often, but not only, of natural origin (biosorbents), e.g., dead and living microorganisms, crab shell, mustard biomass, sawdust, corn silk, *Eucalyptus sheathiana* bark, water lettuce dry biomass, Moringa or *Sophora japonica* pods, herbaceous plants or pine bark algae, microorganisms, coffee and tea grounds, yeasts, nut shells, sawdust, rice husks, or citrus peels [1,13,14,15,16,17,18,19,20,21,22,23,24,25,26,27,28,29,30,31,32,33,34,35,36,37,38,39,40]. Since most of them are in the form of fine or irregular particles, their usage in column processes, as preferred in the industry, or even separation from purified solution is difficult or impossible. The same problem occurs in the case of material made from modified waste PMMA in the form of very fine powder [41]. However, despite the very good sorption capacities towards zinc(II) ions, and the proecological and economical advantage of reusing the waste materials, the sorbent form did not allow one to perform column studies. The solution may be to immobilize the fine particles into a matrix, which may be easily formed into beads. For this purpose pectin [42], alginate [43,44,45] or chitosan [46,47,48] may be used, which are fully biodegradable, have sorption capacities on their own, and, in the case of first two polysaccharides, may be easily crosslinked with calcium ions to form the round beads. Pectin may be recognized as a byproduct from fruit (citrus or apple peels) and vegetable (sugar-beet) industry. Therefore, its usage is in line with the principles of “green chemistry”.

The aim of this work was to prepare and characterize new hybrid sorbent, containing modified waste poly(methyl methacrylate) immobilized in pectin and crosslinked with calcium ions. The material was then studied for zinc ion removal. Besides the sorption kinetics and equilibrium batch studies, a very important and often passed over issue—desorption—was investigated. The efficiency of zinc sorption-desorption cycle was also tested in an industrially-preferred column process. Blending these two materials allows one to apply a PMMA-based sorbent in dynamic system and should display increased sorption capacity compared to pectin alone sorbent, since the sorption capacity of sorbent made from waste PMMA is twice as high as that of the polysaccharide matrix. Additionally, both materials (PMMA and pectin) are generally of waste origin, thus their usage is proecological and helps with waste management. 

## 2. Results and Discussion

### 2.1. Sorbent Preparation and Characterization

Preliminary studies showed that it is possible to introduce about 25% of modified PMMA (MPMMA) into a pectin matrix to obtain stable, round beads. Higher doses of this additive resulted in difficulties in instilling pectin-MPMMA mixture during sorbent preparation and obtaining beads which may be easily cracked when manipulated. 

**Figure 1:**
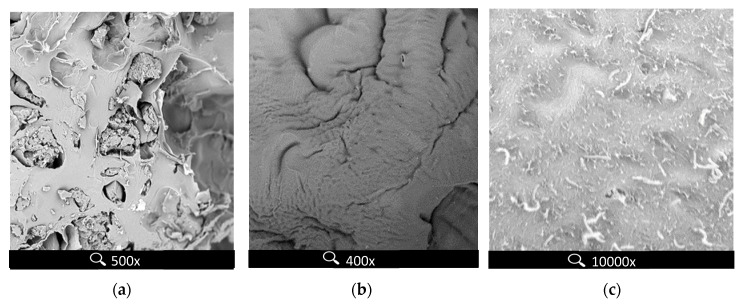
SEM micrographs of inside of the (**a**) hybrid P + MPMMA beads and (**b**) sole pectin beads (**c**) hybrid P + MPMMA beads (outside).

The SEM micrographs of the inside of the beads (Figure 1) show the difference between homogenous and dense structure of sole pectin sorbent (Figure 1b) and laxer hybrid sorbent structure (Figure 1a), where particles of MPMMA are entrapped in the polysaccharide matrix. The outer part of hybrid beads is denser, what prevents the loss of the MPMMA additive from the grains (Figure 1c). The swelling index, determined as the ratio of swollen sorbent mass to mass of sorbent in dry form, is similar for both studied sorbents (Table 1). However, in the case of the hybrid sorbent, swelling of the beads in water is almost the same as in the zinc solution. This is a favorable property, especially in column processes, because no changes in bed volume should happen during washing, sorption or desorption steps.

### 2.2. Sorption—Effect of Sorbent Dose

**Figure 2:**
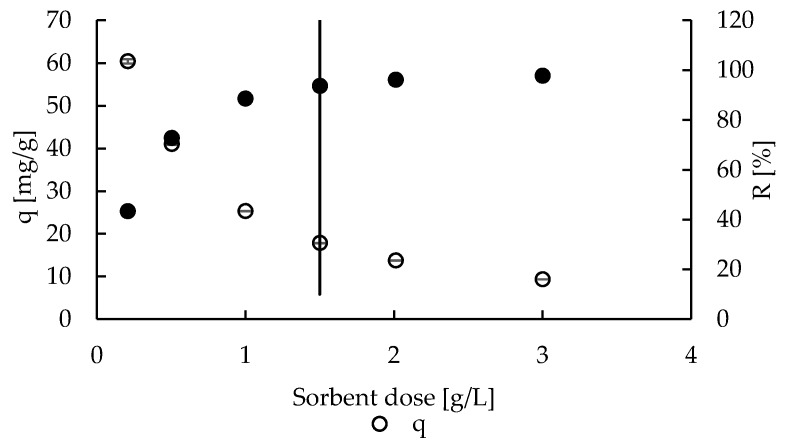
The effect of P + MPMMA sorbent dose on sorption capacity (q) and zinc(II) ions removal percentage (R) (number of replicates *n* = 3, error bars – standard deviations).

Two parameters were taken into account to choose the sorbent dose for further studies—sorption capacity and ions removal percentage. The effect of sorbent dose on these parameters is shown in Figure 2. The highest sorption capacity (ca. 65 mg/g) is obtained by applying the lowest sorbent dose (0.2 g/L), thus the excess of zinc ions is high enough to fill all accessible active sites. Simultaneously, this excess of ions is a reason of low zinc(II) removal percentage in such conditions (ca. 40%). Increasing the sorbent dose to 3 g/L, thus decreasing or leveling the excess of zinc ions to number of active sites, significant decreasing in sorption capacity (to about 10 mg/g) and increasing in removal percentage (to above 95%) is observed. In such conditions, some active sites may be unsaturated, what caused the mentioned sorption capacity decrease. The sorbent dose chosen for the further studies is indicated by vertical line in Figure 2. It is 1.5 g/L—in such conditions the percentage of Zn(II) removal is still above 90%, but the sorption capacity is as high as 20 mg/g.

### 2.3. Sorption—Effect of pH

**Figure 3:**
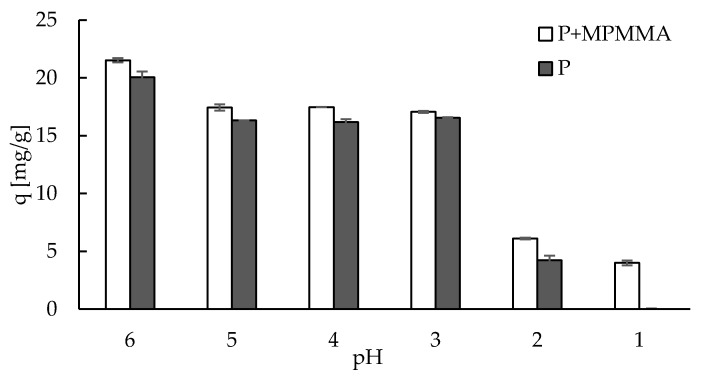
The effect of pH on sorption capacity (q) towards zinc(II) ions (number of replicates *n* = 3, error bars—standard deviations).

The effect of pH on sorption capacity towards Zn(II) ions of the studied material is shown in Figure 3. The hybrid sorbent, similarly to pectin alone sorbent, showed very good or even greater affinity to Zn(II) ions in a broad pH range (3–6). It may be noticed that the sorption capacity of P + MPMMA did not drop significantly in pH = 3 as it was in the case of pure MPMMA material (q < 10 mg/g) [41]. Taking into account that hybrid material contained about 25% of MPMMA, the decrease in sorption capacities should be visible in pH = 3. Therefore, some synergistic effect (P and MPMMA) might have happened. The highest sorption capacity was obtained in pH = 6, thus zinc(II) solution of pH = 6 was applied in further studies. 

### 2.4. Sorption—Kinetics 

**Figure 4:**
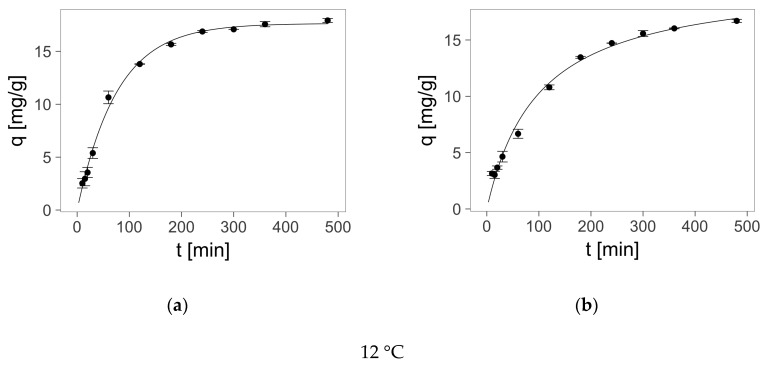

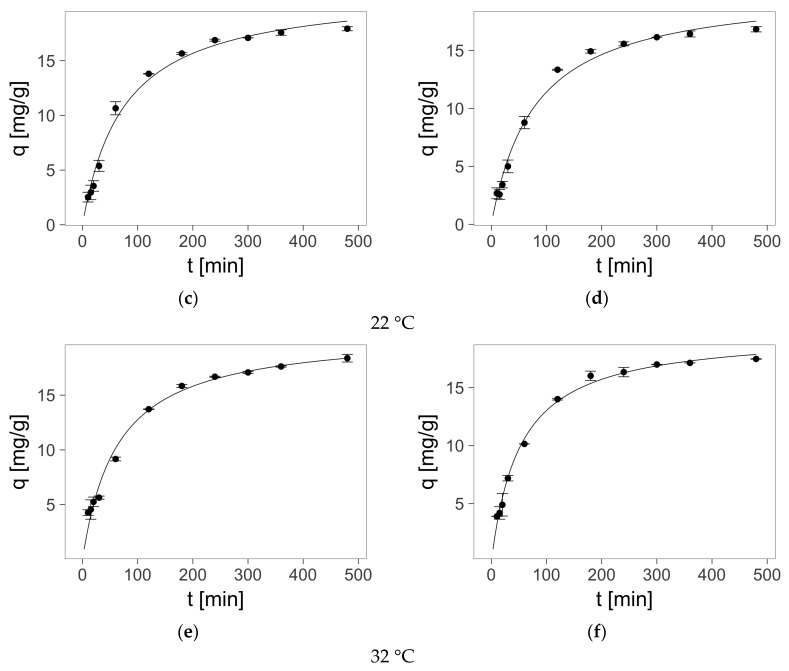
Pseudo second order kinetics of zinc(II) ions sorption in various temperatures for hybrid P + MPMMA sorbent (**a**,**c**,**e**) and for sole P sorbent (**b**,**d**,**f**) (number of replicates *n* = 3, error bars—standard deviations).

In order to describe and compare the sorption rate of the hybrid and the sole pectin sorbents, the parameters of pseudo-first-order and pseudo-second-order kinetic models were estimated based on the results of the Zn(II) sorption kinetics experiments. The parameters and kinetic curves obtained at various temperatures for both sorbents are depicted in Table 2 and in Figure 4, respectively. 

**Figure 5:**
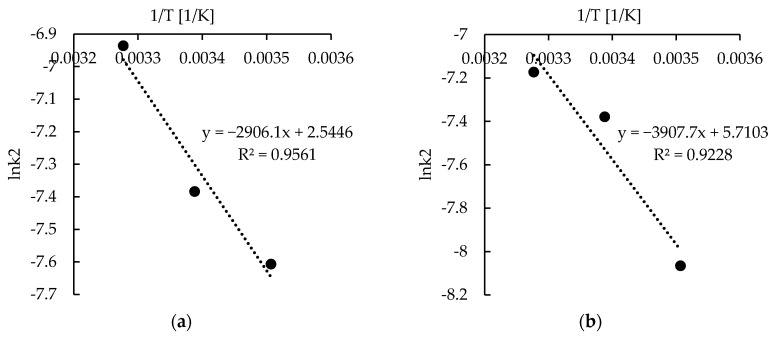
Plot of lnk_2_ versus 1/T for calculation of activation energy of the zinc ions sorption on (**a**) P + MPMMA sorbent and (**b**) sole P sorbent.

At room temperature (22 °C), the sorption rates for P and P + MPMMA materials do not differ significantly and equilibrium is achieved after about 5–6 h. However, almost 90% of maximum reached sorption capacity is achieved within the first 3 h. The rate constant of the adsorption process increases with increasing temperature. This suggests that the sorption of zinc(II) ions on the studied materials is an endothermic process. However, the results obtained at 12 °C and 32 °C showed opposite sensitivity of the studied sorbents to the temperature change. In the case of the hybrid sorbent, increasing the temperature by 10 degrees resulted in only a slight (ca. 20%) increase in sorption rate represented by the rate constant (k_2_) from the kinetic model. The same change of temperature in the case of the only pectin sorbent resulted in a more serious sorption rate change (above 50%). Inversely, lowering the temperature by 10 degrees induces the greater changes in sorption rate of P + MPMMA sorbent—the rate constant decreases by half. The sorption rate constant of P material drops in these conditions only by 20%. The amount of zinc (II) ions adsorbed at equilibrium does not vary significantly with temperature. Additionally, activation energy of the Zn(II) ions sorption on studied materials was determined. The ln k_2_ was plotted against 1/T and straight line was fitted (Figure 5) with the correlation coefficient of 0.923 and 0.956 for P-MPMMA and P sorbents respectively. The activation energy, calculated as the slope of the fitted curve multiplied by (–R), equaled 24.2 and 32.5 kJ/mol for sole and hybrid pectin-based sorbent respectively. These values are a measure of the energy barrier during sorption, which lie in the range of the activation energy of chemisorption processes (8.4–83.7 kJ/mol) [49] . Therefore it may prove the main mechanism of sorption to be chemisorption (ion-exchange Ca^2+^–Zn^2+^) [41,50].

### 2.5. Sorption—Isotherms 

**Figure 6:**
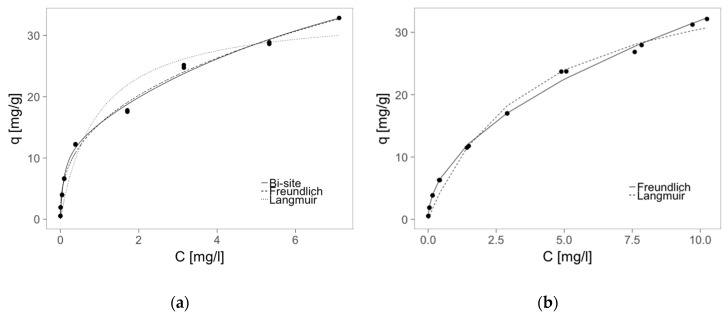
Adsorption isotherms of zinc on (**a**) P + MPMMA and (**b**) P sorbent.

Based on the results of the Zn(II) equilibrium sorption experiments, the parameters of two elementary isotherms were estimated and shown in Table 3. The best fit lines for Langmuir and Freundlich model are depicted in Figure 6. 

In the case of the only pectin sorbent, both models fit the experimental data well. The obtained parameters are close to but a little higher from those obtained in our previous study in similar conditions for sole pectin biosorbent [50]. The pectin as raw material may vary from one source to another, therefore the comparison studies should be performed using sole pectin beads made from the same portion of raw materials, which is used for hybrid sorbent synthesis. The affinity of zinc(II) ions to hybrid sorbent is significantly higher than for pectin alone sorbent, which is reflected by higher B and K_f_ or n value of parameters from Langmuir and Freundlich models, respectively. This is due to the MPMMA additive, which as a simple powder has greater sorption capacity as well as other sorption parameters (B, K_f_ and n) than pectin sorbent [41]. Only sorption capacity (q_m_) of P + MPMMA calculated form Langmuir isotherm is a little lower than for P sorbent, despite that the MPMMA additive as sorbent has greater sorption capacity towards Zn(II) ions (about 80 mg/g) [41], thus should enhance the q_m_ of hybrid one. It may be caused by worse fit of this model to experimental data (lower correlation coefficient: 0.973) than Freundlich one (R^2^ = 0.996). Taking into account that hybrid sorbent contains two different sorbents (modified waste poly(methyl methacrylate) and pectin), thus two types of sorption sites with different adsorption energy may coexist, the parameters of the Langmuir bi-site model [51] were also estimated (Table 3). A high correlation coefficient (0.996) proved this model describes sorption on hybrid sorbent much better than mono-site Langmuir equation. The estimated sorption capacities q_m1_ and q_m2_ were on the same order of magnitude, therefore coexistence of two significant sorption sites of different adsorption energy (B1, B2) was confirmed. Additionally, the sum of the capacities was higher than capacity of sole pectin sorbent, which was in agreement with our hypothesis. 

### 2.6. Desorption—Equilibrium 

**Figure 7:**
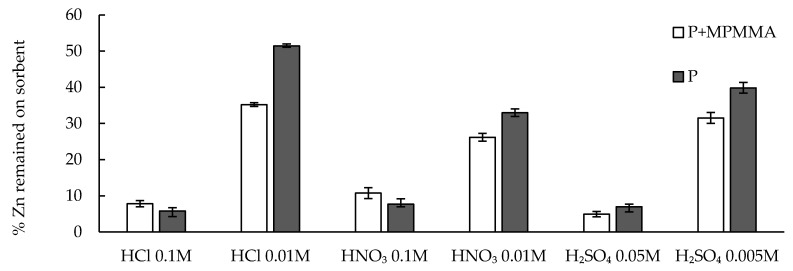
Comparison of zinc(II) ions desorption from P + MPMMA and P sorbents using hydrochloric, nitric and sulfuric acids of various concentrations (number of replicates *n* = 3, error bars—standard deviations).

The main disadvantage of sorption processes is the necessity of loaded sorbent management. However, majority of authors pass over this problem, especially in the case of biosorption studies. The main fates of the used sorbent management are burning the material with energy and metal oxides recovery or desorption and regeneration of material to be ready for reuse. These steps are very important issues in the sorption processes and they may determine their economic and proecological aspects. In this work, the possibility of zinc ion removal from the saturated hybrid sorbent using hydrochloric, nitric and sulfuric acids solutions of various concentrations was investigated. The desorption results are presented as the percent of Zn(II) ions remaining on the sorbent after stripping with acid solution (Figure 7). 

In order to compare the average results obtained for various acids some statistical tests were carried out. First TEST.F (Microsoft Excel, Microsoft Inc., Redmond, WA, USA), which corresponds to the F-Snedecor test (α = 0.05, *n* = 3), was performed to compare the variances. This allowed employing the proper T.TEST parameter which returns the probability (p) associated with a Student’s *t*-Test (α = 0.05, *n* = 3) for comparison of the two average results. Statistical analysis proved for both sorbents there were no significant differences between results obtained for various 0.1 M (in conversion to H^+^ concentration) acids. The “*p*” value were above significant level (0.05) in the case of all acid pairs: 0.1 M HCl–0.1 M HNO_3_ (P: 0.272, P + MPMMA: 0.184), 0.1 M HCl–0.05 M H_2_SO_4_ (P: 0.516, P + MPMMA: 0.094), 0.1 M HNO_3_–0.05 M H_2_SO_4_ (P: 0.0536, P + MPMMA: 0.605). Similarly the results for pairs of the same acids but of various concentration were compared. In this case the statistical analysis proved that differences between averages calculated for 0.01 and 0.1 M acids solution were significant—the “*p*” values were below significant level (0.05) for the pairs as follows: 0.01 M HCl–0.1 M HCl (P: 0.00063, P + MPMMA: 0.00069), 0.005 M H_2_SO_4_–0.05 M H_2_SO_4_ (P: 0.0023, P + MPMMA: 0.0019), 0.01 M HNO_3_–0.1 M HNO_3_ (P: 0.0068, P + MPMMA: 0.0012). Generally, it may be stated that more concentrated acid solutions remove zinc ions from studied materials more efficiently than diluted ones. Independently of the kind of acid used, the desorption was at least 90% and similar for both types of beads. More diluted acid solutions were able to strip up to 74% of the Zn(II) ions retained on the sorbent. However, this process seems to be more effective in the case of our hybrid material. The presented results prove that 0.1 M solutions of all studied acids (in conversion to proton concentration) are suitable for removal of zinc(II) ions from the polymer-pectin beads, therefore the kind of acid may be chosen taking account further metal ion solution processing.

### 2.7. Sorption—Breakthrough Curves 

**Figure 8:**
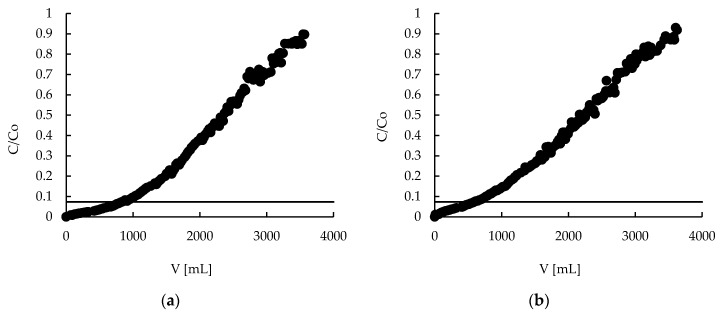
Zinc(II) ions breakthrough curves on (**a**) hybrid P + MPMMA sorbent and (**b**) only P sorbent.

Column studies are very important from an industrial point of view. Column processes are desirable in the industry because are more efficient, easy to automate and less space-consuming than the batch ones. Thanks to the pectin matrix, powdered MPMMA sorbent was converted into a hybrid material, which was in the form of beads suitable for use in column experiments. The obtained breakthrough curves for both sorbents (Figure 8) are of a specific shape—there is no typical breakpoint. The concentration of Zn(II) ions increases in the effluent firstly exponentially and then monotonically to achieve its initial value (c/c_0_ = 1). On the face of it, both curves are very similar, but after careful analysis it may be seen that hybrid sorbent in the first stage of process (e.g., first 500 mL) removes zinc(II) ions more deeply. This is very important, because this range is the practical working range. Taking into account the shape of the breakthrough curves and the permissible limits for zinc in wastewater introduced into environment (1.5–2.61 mg/L) [4,5,6], the end point of column work was established to be when the concentration of zinc in the effluent achieves 2.61 mg/L. In this case, when the initial concentration of zinc equaled 35.5 mg/L, this value corresponds to c/c_0_ = 0.073 (marked as a horizontal line in the figures). Therefore, all further column studies were performed until the c/c_0_ achieved 0.073. 

### 2.8. Sorption—Effect of Flow Rate 

**Figure 9:**
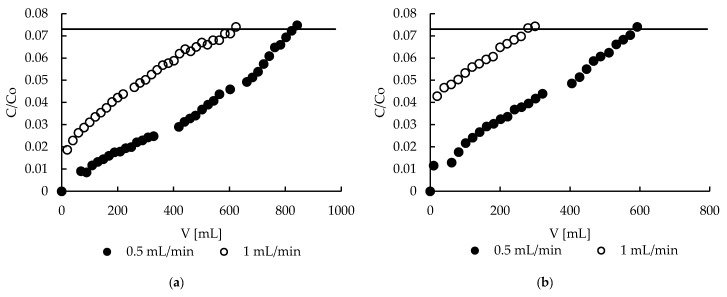
Effect of flow rate on zinc(II) ion removal on (**a**) hybrid P + MPMMA sorbent and (**b**) sole P sorbent.

The studies on the effect of flow rate on zinc(II) ions removal on column filled with pectin-based materials showed that increasing the flow rate from 0.5 to 1 mL/min resulted in a drop of solution purification (Figure 9). However, the effect depends on the sorbent used. In the case of hybrid sorbent, the increase of flow rate decreases amount of purified solution only of about 25%. In the case of the only pectin sorbent this decrease equals about 50%. The results confirmed presented in batch studies (Langmuir bi-site) higher sorption capacity of hybrid sorbent. The sorbent containing MPMMA allows for purification of greater amount of zinc solution to desirable, permitted by law, level. In the applied conditions, it is above 0.83 L vs. about 0.60 L of solution purified by P + MPMMA and P materials respectively. 

### 2.9. Sorption—Effect of Bed Height

**Figure 10:**
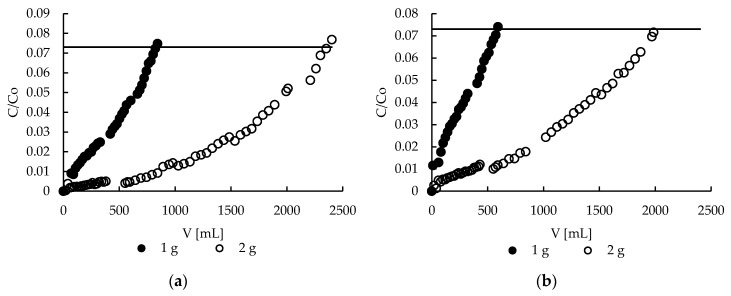
Effect of bed height on zinc(II) ions removal on (**a**) hybrid P + MPMMA sorbent and (**b**) only P sorbent.

During the studies on effect of bed height on zinc(II) ions removal on column filled with pectin-based materials two various flow rates were applied to ensure the same residence time of the ions in the column in each case (0.5 mL/min for 1 g of sorbent (dry form) and 1 mL/min for 2 g of sorbent (dry form), the column diameter was always 1.5 cm). The results presented in Figure 10 show that by doubling the amount of sorbent, and thus the bed height, more than double the amount of purified solution is obtained. It is above 2.3 L and about 2.0 L vs about 0.83 L and about 0.60 L for 2 and 1 g of hybrid and sole pectin sorbents, respectively. 

Therefore, it may be stated that an increase of the bed height resulted in an increase of zinc removal efficiency in the studied range. Independently of the bed height the sorbent with MPMMA additive purified a greater (by about 15–30%) amount of Zn(II) solution. As mentioned above the MPMMA sorbent alone has greater sorption capacity (80 mg/g) [41] than pectin one. Therefore, this sorption efficiency increase is probably due to the addition of MPMMA.

### 2.10. Desorption—Effect of Flow Rate

**Figure 11:**
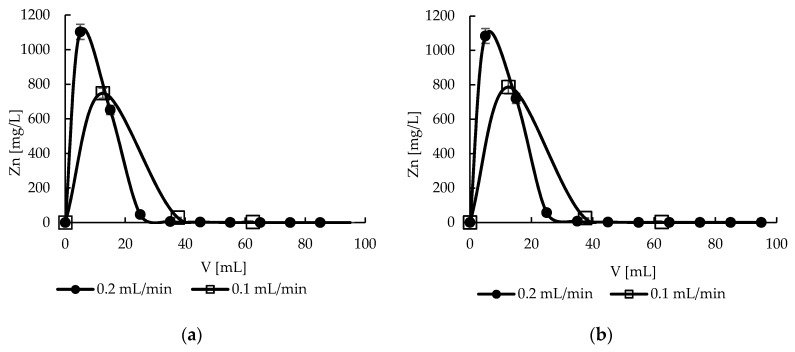
Effect of flow rate on zinc(II) ions stripping from (**a**) hybrid P + MPMMA sorbent and (**b**) only P sorbent (number of replicates *n* = 3, error bars—standard deviations).

As mentioned above, the desorption of ions retained on the material is very important issue in the sorption processes and it may determine their economic and proecological aspects. The stripping of metal ions from sorbent should be complete and as little as possible stripping solution should be used to concentrate the element to the greatest extent. Taking into account similar efficiency of various 0.1 M acids solution, proved by statistical analysis in Section 2.6, 0.1 M solution of nitric acid was selected for column desorption studies. Applying the flow rate 0.2 mL/min, fast elution of zinc(II) ions was obtained with first 20 mL of eluent (Figure 11). It was almost identical for both sorbents. Employing the results from column sorption studies it may be calculated that Zn(II) ions were concentrated above 30 and ca. 25 times using hybrid and sole pectin sorbents, respectively. Decreasing the flow rate to 0.1 mL/min did not improve or worsen the stripping results.

### 2.11. Desorption—Effect of Bed Height

**Figure 12:**
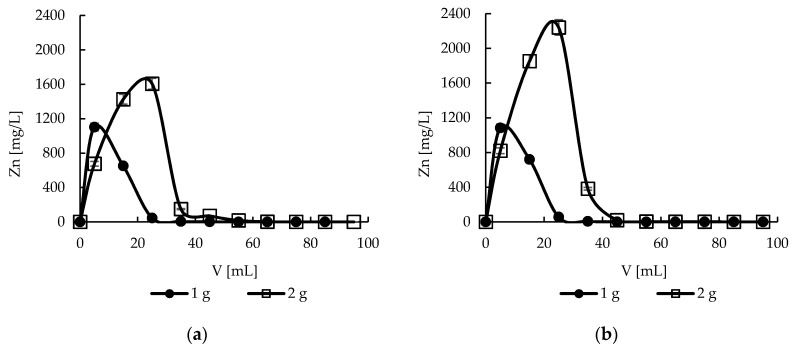
Effect of bed height on zinc(II) ions stripping from (**a**) hybrid P + MPMMA sorbent and (**b**) only P sorbent (number of replicates *n* = 3, error bars—standard deviations).

Doubling the amount of sorbent, and thus the bed height, double the amount (ca. 40 mL) of eluent is necessary for stripping the zinc(II) ions from sorbents (Figure 12). Taking into account these results and results from sorption experiments the concentration of zinc ions in the case of a higher bed was calculated. It was about 58 and 50 times for hybrid and sole pectin sorbent respectively (Figure 12). Such concentrated solutions may be further processed and finally the pure metal may be isolated e.g., by electrolysis.

### 2.12. Sorption—Desorption—Mass Balance of the Whole Process

Additionally, the efficiency of the desorption step was evaluated by calculating the Zn mass balance of the sorption-desorption cycle (Table 4). The good recovery of the element proved the stripping method to be applicable for Zn(II) removal from studied sorbents. 

## 3. Materials and Methods 

### 3.1. Materials

Amide pectin (type NECJ A20) of amidation degree 19.9% and esterification degree 30.2% was supplied by C&G Spółka z o.o. (Jasło, Poland). The previously obtained and characterized sorbent—modified waste poly(methyl methacrylate) crosslinked with calcium ions (MPMMA) [41] in the form of powder (<200 µm) was used. The following reagents were also used: sodium hydroxide (Avantor, Gliwice, Poland), concentrated nitric, sulfuric and hydrochloric acids (all “Suprapur” from Merck, Darmstadt, Germany), zinc nitrate (Avantor), standard solution of zinc (1000 mg/L, Merck). Deionized water was prepared using Millipore Elix 10 system (Milipore SAS, Molsheim, France).

The 3% solution of pectin (P) was prepared by shaking the appropriate amount of pectin in deionized water at 50 °C using a thermostated shaker (Incu-Shaker, Benchmark, Sayreville, NJ, USA). Next the MPMMA (P:MPMMA = 3:1) was added and shaken at 50 °C for 2 h. The mixture was next slowly instilled with a peristaltic pump into a continuously mixed 1 M calcium chloride solution (~4 °C). As the result, round beads of ca. 3 mm diameter were obtained. As we tested previously [52], each 200 g of pectin-based solution required the use of at least 1 L of cold 1 M CaCl_2_ solution. The hybrid sorbent was then kept in the mother solution at about 4 °C for at least 24 h. After that it was filtered (medium speed filter paper) and washed until complete removal of chloride ions. Next, the material was dried at 35 °C for at least 24 h to obtain beige xerogel of diameter ca. 1 mm and moisture content of ca. 10% (determined at 105 °C). Simultaneously, the sorbent containing only pectin was prepared for a comparison study. The physical properties of hybrid and only pectin sorbent such as the swelling index in water and zinc solution were then investigated. 

### 3.2. Analytical Method

The determination of zinc(II) in all solutions was performed by the ICP-AES method (Varian 710-ES ICP atomic emission spectrometer, Varian, Palo Alto, CA, USA) using the following parameters: RF power 1.0 kW, plasma flow 15 L/min, auxiliary flow 1.5 L/min, nebulizer pressure 200 kPa, pump rate 15 rpm, emission lines: λ_Zn_ = 328.233 nm, λ_Zn_ = 334.502 nm, λ_Zn_ = 472.215 nm. The concentration of zinc was calculated using the calibration curve method in the range of 0.1–40 mg/L. Swollen and lyophilized (Christ Alpha 1-2 LDplus, Martin Christ Gefriertrocknungsanlagen GmbH, Osterode am Harz, Germany) materials were analyzed utilizing scanning electron microscopy (Phenom Pro Desktop SEM, Eindhoven, The Netherlands).

### 3.3. Sorption—General Procedure for Batch Studies

A proper amount of weighed sorbent (dry form) was placed in plastic vessels and solution of Zn(NO_3_)_2_ of pH = 6 was added. Next, the closed vessels were shaken for 24 h at room temperature (22 ± 1 °C). After that, the sample was filtered (medium speed filter paper) and, if necessary, diluted properly to enable the determination of Zn concentration by the ICP-AES method. The concentration of the initial solution was also determined in each case. The most important sorption parameter, sorption capacity of zinc(II) ions on studied pectin-based sorbents (mg/g), was calculated as follows:

(1) $$q=\frac{\left(c_{0}-c\right)\times V}{m}$$

where: $c_{0}$—the initial concentration of zinc(II) ions in the solution (mg/L), c—the final concentration of zinc(II) ions in the solution (mg/L), V—the volume of the solution (L), and m—the mass of the sorbent in dry form (g).

### 3.4. Sorption—Effect of Sorbent Dose

The studies were performed according to general procedure, but various sample weights (0.01–0.2 g) were contacted with 50 mL of Zn(II) solution of initial concentration about 30 mg/L.

### 3.5. Sorption—Effect of pH

The studies were performed according to the general procedure, but 15 mg of sorbent was contacted with 10 mL of Zn(II) solution of initial concentration about 30 mg/L and various pH (1–6). The pH of the solutions was adjusted with nitric acid or sodium hydroxide. 

### 3.6. Sorption—Kinetics 

The studies were performed according to general procedure, but 0.75 g of sorbent was contacted with 500 mL of Zn(II) solution of initial concentration about 30 mg/L at various temperatures (T = 12 °C; T = 22 °C; T = 32 °C). After proper time (1, 3, 10, 15, 20, 30, 60, 120, 180, 240, 300, 360, and 480 min) the samples of 0.1 mL were collected and diluted in volumetric flasks (2 mL) with distilled water. Since the solution lost during whole experiment caused by sample taking was only 0.26%, the change of solution volume during experiment was neglected.

Based on the results of the Zn(II) sorption kinetics experiments, the parameters of pseudo-first-order and pseudo-second-order kinetic models were estimated using the formulas:

The pseudo-first-order equation [53,54]:

q_t_ = q_m_ × (1 − e ^−k^_1_^× t^) (2)

The pseudo-second-order equation [54]:

q_t_ = (k_2_ × q_m_^2^ × t)/(1 + (k_2_ × q_m_ × t)) (3)

where: q_m_—the zinc(II) ions adsorbed on one gram of material at equilibrium (adsorption capacity) (mg/g), q_t_—the zinc(II) ions adsorbed on one gram of material at time “t” (mg/g), k_1_—the rate constant of pseudo-first order adsorption model (1/min), k_2_—the rate constant of pseudo-second order adsorption model (g/(mg·min)), and t—the time (min).

Additionally, the activation energy of the Zn(II) ions sorption on studied materials was determined using the Arrhenius equation [55]:

ln k_2_ = ln A − E_a_/R·T (4)

where: k_2_—the rate constant at given temperature obtained from the pseudo-second-order kinetics (g/mg·min), E_a_—the Arrhenius activation energy (J/mol), A—the Arrhenius factor (g/(mg·min)), R—the gas constant (8.314 (J/(mol·K)), and T—the solution temperature (K). The ln k_2_ was plotted against 1/T and straight line was fitted. The activation energy was calculated as the slope of the fitted curve multiplied by (–R). 

### 3.7. Sorption—Isotherms

The studies were performed according to general procedure, but 15 mg of sorbent was contacted with 10 mL of Zn(II) solution of various initial concentration (about 1, 3, 6, 10, 20, 30, 40, 50 and 60 mg/L). 

Based on the results of the Zn(II) equilibrium sorption experiments, the parameters of two elementary isotherms were estimated using the formulas:

Langmuir isotherm [56]:

q = (q_m_ × B × c)/(1 + B × c) (5)

Freundlich isotherm [56]:

q = K_f_ × c^1/n^ (6)

where: q—the zinc(II) ions adsorbed on one gram of material at equilibrium (sorption capacity) (mg/g), q_m_—the maximum adsorption capacity (mg/g), B—the equilibrium constant that corresponds to the adsorption energy (L/mg), c—the equilibrium concentration of zinc(II) ions in the solution (mg/L), K_f_—((mg/g)(L/mg)^1/n^) corresponds to the relative adsorption capacity, and n—corresponds to the adsorption intensity of the sorbent.

Additionally, the parameters of Langmuir bi-site model [51] were estimated using the formula:

q = ((q_m1_ × B_1_ × c)/(1 + B_1_ × c)) + ((q_m2_ × B_2_ × c)/(1 + B_2_ × c)) (7)

where: q_m1_, q_m2_—adsorption capacities for different sorption sites (mg/g), and B_1_, B_2_—equilibrium constants for different sorption sites that correspond to the adsorption energy (L/mg).

The kinetic as well as equilibrium model parameters and their errors were estimated by fitting the equation in its non-linearized forms to the all the data points collected during the respective experiment. The calculations were performed using the modified Levenberg-Marquardt algorithm for non-linear least square estimations [57], as implemented in the minpack.lm package of the GNU R statistical software [58]. Estimated parameters were judged as significant if their calculated *p*-value was below 1%.

### 3.8. Desorption—Equilibrium 

The first step was to fill the material with Zn(II) ions according to general procedure, but 15 mg of sorbent was contacted with 10 mL of Zn(II) solution of an initial concentration of about 30 mg/L. After 24 h the sorbents were filtered, gently dried with paper towel, and contacted in the plastic vessels with 5 mL of acid solution (0.01 M HNO_3_, 0.1 M HNO_3_, 0.01 M HCl, 0.1 M HCl, 0.005 M H_2_SO_4_, 0.05 M H_2_SO_4_) for 24 h at room temperature (22 ± 1 °C). After that solution samples from sorption and desorption steps were analyzed using ICP-AES method. The efficiency of desorption was expressed as the amount of zinc(II) ions remained on the pectin-based sorbent (in mass percent) after desorption step. It was calculated by dividing the amount of zinc(II) ions found in the acid solution after desorption by the amount of zinc(II) ions sorbed on the beads during sorption step.

### 3.9. Sorption—General Procedure for Column Studies 

The feed solution (Zn(II) solution of initial concentration about 30 mg/L and pH = 6) was supplied to the column (internal diameter 15 mm) containing sorbent in wet form using a peristaltic pump at constant flow rate and room temperature (22 ± 1 °C). The effluent from the column was collected into fractions of 20 mL. Zn(II) concentration in each sample was determined using ICP-AES method. The breakthrough curves were obtained by plotting metal concentration change (fraction of initial concentration (c/c_0_)) against the volume of collected effluent (V (mL)). 

The whole breakthrough curves were obtained according to the general procedure, but 1 g of sorbent (dry form) and 0.5 mL/min flow rate were applied. The studies on effect of flow rate were performed according to general procedure, but 1 g of sorbent (dry form) and various flow rate (0.5 and 1 mL/min) were applied. The studies on effect of bed height were performed according to general procedure, but 1 g of sorbent (dry form) and 0.5 mL/min flow rate or 2 g of sorbent (dry form) and 1 mL/min flow rate were applied. 

### 3.10. Desorption—General Procedure for Column Studies 

The first step was to load the material with Zn(II) ions according to the general procedure (Section 3.9). Next, a 0.1 M HNO_3_ solution was supplied to the column using a peristaltic pump at a constant flow rate. The effluent from the column was collected in fractions of 10 mL. Zn(II) concentration in samples from sorption and desorption experiments was determined using ICP-AES method. The elution curves of desorption were obtained as dependence of concentration of zinc(II) ions in collected fraction (c (mg/L)) on volume of the collected effluent (V (mL)).

The studies of the flow rate effect were performed according to general procedure, but 1 g of sorbent (dry form) and various flow rate (0.1 and 0.2 mL/min) were applied. The studies on effect of bed height were performed according to general procedure, but 1 g of sorbent (dry form) and 0.2 mL/min flow rate or 2 g of sorbent (dry form) and 0.4 mL/min flow rate were applied.

All sorption and desorption experiments were done in triplicate, so each result is an average value from three independent trials. The exceptions are isotherms and breakthrough curves—in these cases each experimental point was duplicated and all results were placed in the figures instead of average values. 

## 4. Conclusions

Our studies have shown that pectin may be a good matrix for powdered PMMA-based sorbent immobilization. The main advantages of such a matrix are its biodegradability, its own inherent sorption capacity and simplicity of shape formation. As a result, round beads of sorbent were obtained (P:MPMMA = 3:1), which were suitable for—very important from an industrial point of view—column studies. These studies showed our hybrid sorbent to be, depending on the conditions, 15–30% more efficient for zinc(II) ion removal than pectin alone sorbent. This is probably due to greater sorption capacity of MPMMA alone sorbent than pectin ones. Despite the higher sorption affinity of zinc(II) ions to the hybrid sorbent, reflected by higher values of its Langmuir and Freundlich model parameters, the stripping of these ions from P + MPMMA beads using diluted (0.1 M) solutions of mineral acids is as effective as in the case of only pectin sorbent. The mass balance calculated for the column studies showed about 100% recovery of zinc in sorption-desorption cycles. Applying the hybrid sorbent under the studied conditions it is possible to purify water to the Zn level permitted by law and concentrate Zn(II) ions by about 60-fold. These promising results suggest further work on optimization of the parameters of this process, especially considering that both materials (MPMMA and pectin) are generally of waste origin, thus their usage is proecological and helps waste substance management.

## Figures and Tables

**Table 1:** Swelling index of hybrid P + MPMMA and sole P sorbets in deionized water and in zinc solution.

Sorbent	Swelling Index in Water	Swelling Index in Zinc Solution
P + MPMMA	2.50 ± 0.03	2.43 ± 0.03
P	2.30 ± 0.01	2.50 ± 0.01

**Table 2:** Estimated parameters of pseudo-first and pseudo-second order adsorption kinetics model of zinc(II) ions sorption on hybrid P + MPMMA and only P sorbent.

	**Pseudo-First-Order Kinetics for P + MPMMA Sorbent**			**Pseudo-First-Order Kinetics for P Sorbent**		
	**12 °C**	**22 °C**	**32 °C**	**12 °C**	**22 °C**	**32 °C**
**R^2^**	0.992	0.996	0.985	0.991	0.998	0.992
**q_m_ (mg/g)**	17.60 ± 0.80	17.63 ± 0.24	17.51 ± 0.58	16.43 ± 0.61	16.65 ± 0.18	16.94 ± 0.37
**k_1_·10^3^ (1/min)**	7.53 ± 0.94	13.19 ± 0.65	14.22 ± 1.71	9.86 ± 1.16	12.55 ± 0.48	16.98 ± 1.41
	**Pseudo-Second-Order Kinetics for P + MPMMA Sorbent**			**Pseudo-Second-Order Kinetics for P Sorbent**		
	**12 °C**	**22 °C**	**32 °C**	**12 °C**	**22 °C**	**32 °C**
**R^2^**	0.990	0.992	0.986	0.989	0.992	0.992
**q_m_ (mg/g)**	22.69 ± 1.35	21.50 ± 0.68	20.77 ± 0.78	20.39 ± 0.96	20.41 ± 0.64	19.80 ± 0.48
**k_2_·10^4^ (g/mg∙min)**	3.14 ± 0.67	6.24 ± 0.83	7.67 ± 1.27	4.97 ± 0.92	6.21 ± 0.82	9.72 ± 1.07

**Table 3:** Calculated parameters of Langmuir, Freundlich and bi-site Langmuir isotherms, of Zn(II) ions sorption on P + MPMMA and P sorbents.

**Calculated Parameters of Langmuir Isotherm**			**Calculated Parameters of Freundlich Isotherm**		
	**P + MPMMA**	**P**		**P + MPMMA**	**P**
**q_m_ (mg/g)**	35.03 ± 2.21	42.17 ± 2.43	**K_f_ ((mg/g)(L/mg)^1/n^)**	15.46 ± 0.27	9.92 ± 0.20
**B (L/mg)**	0.93 ± 0.22	0.26 ± 0.04	**n**	2.61 ± 0.07	1.97 ± 0.04
**R^2^**	0.973	0.992	**R^2^**	0.996	0.98
**Calculated Parameters of Bi-Site Langmuir Isotherm**					
**P + MPMMA**					
**q_m1_ (mg/g)**			50.2 ± 11.5		
**q_m2_ (mg/g)**			11.8 ± 1.7		
**B_1_ (L/mg)**			0.10 ± 0.05		
**B_2_ (L/mg)**			12.1 ± 3.9		
**R^2^**			0.996		

**Table 4:** The mass balance of zinc(II) ions sorption-desorption cycle in dynamic studies.

Sorbent	(1) Zn Introduced to the Column (mg)	(2) Zn in Effluent (Sorption Process) (mg)	(3) Zn in Effluent (Desorption Process) (mg)	(4) the Sum of (2) and (3) (mg)	Recovery (%)
**P + MPMMA**	126.58	45.92	82.68	128.61	101.60
**P**	128.67	52.07	78.08	130.15	101.15
